# Supplementary material for: A keystone mutualism underpins resilience of a coastal ecosystem to drought
Source: Nat Commun. 2016 Aug 18;7:12473. doi: 10.1038/ncomms12473 (PMC4992128; doi:10.1038/ncomms12473)
Supplement: Supplementary Information — Supplementary Figures 1-7 and Supplementary Tables 1-3 [file ncomms12473-s1.pdf]

Data type	Start Date	End Date
Latitudinal survey	June 2012	July 2012
Porewater salinity monitoring	May 2013	August 2013
Mussel addition experiment	April 2011	Sept. 2012
Cordgrass recovery monitoring	Dec. 2011	May 2013
Clonal recovery experiment	May 2008	August 2012

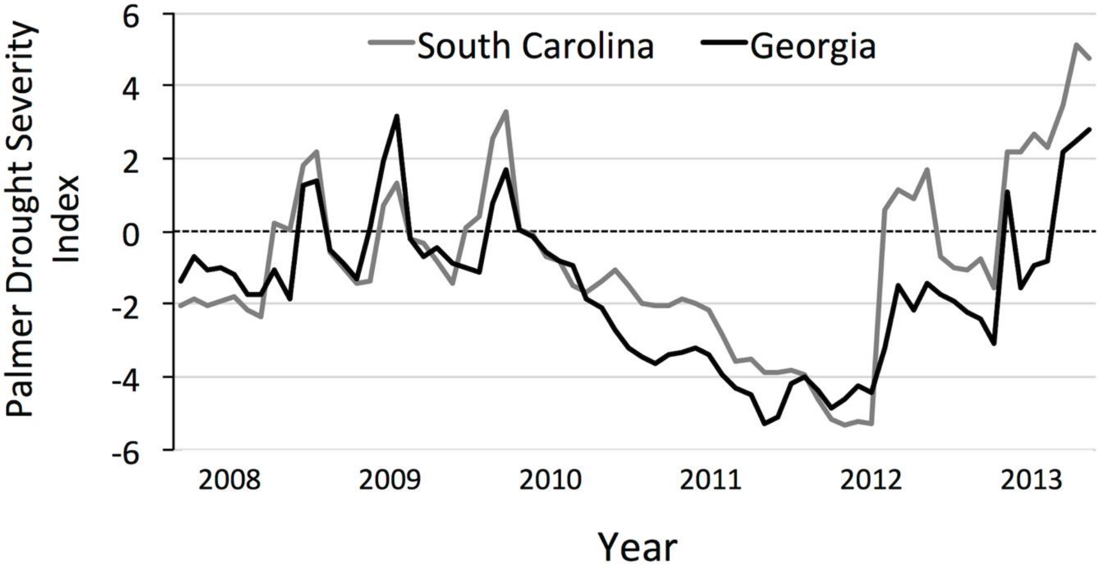

Supplementary Figure 1. Summary of timeframe for experiments and monitoring presented in this study and the Palmer Drought Severity Index (PDSI) for coastal South Carolina (in grey) and Georgia (in black) from 2008, prior to the start of this study, through December 2013. Values that fall below -2 indicate a moderate drought, below -3 a severe drought, and below -4 an extreme drought. Data are from the NOAA National Center for Environmental Information database.

$$P_{\text{Cordgrass resisting drought with mussels}} = \Sigma C_{\text{Mussels}} / (\Sigma C_{\text{Mussels}} + \Sigma D_{\text{Mussels}})$$

$$P_{\text{Cordgrass resisting drought without mussels}} = \Sigma C_{\text{No mussels}} / [\Sigma C_{\text{No mussels}} + (\Sigma A_{\text{Mudflat}} - \Sigma C_{\text{Mussels}} - \Sigma D_{\text{Mussels}})]$$

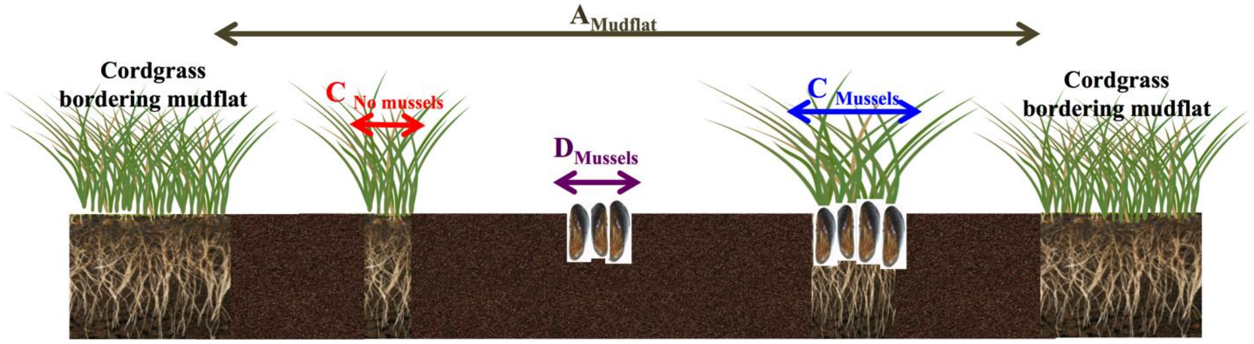

Supplementary Figure 2. Cross-section schematic of a drought-generated die-off and the measurements we collected in our latitudinal survey to calculate the probability of cordgrass resisting the drought when associated with mussels and not associated with mussels, as shown in Figure 1 in the main text. $A_{\text{Die-off}}$ refers to the die-off area; C_{Mussels} refers to the area of a remnant cordgrass patch associated with mussels; $C_{\text{No mussel}}$ refers to the area of a remnant cordgrass patch associated not with mussels; and D_{Mussel} refers to the area of a cordgrass patch that died in association with mussels. Data from patches of each type and from all mudflats observed at each site were summed to calculate the probability of cordgrass resisting the drought with and without mussels.

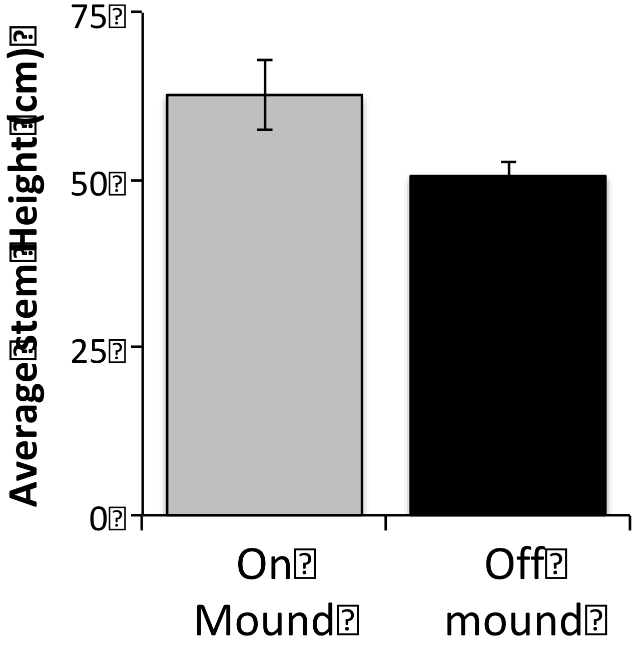

Supplementary Figure 3. Cordgrass stem height within the elevated pseudofeces layer and in the adjacent marsh platform. Data are the mean ± SEM of stem height measures collected in 3 replicate plots.

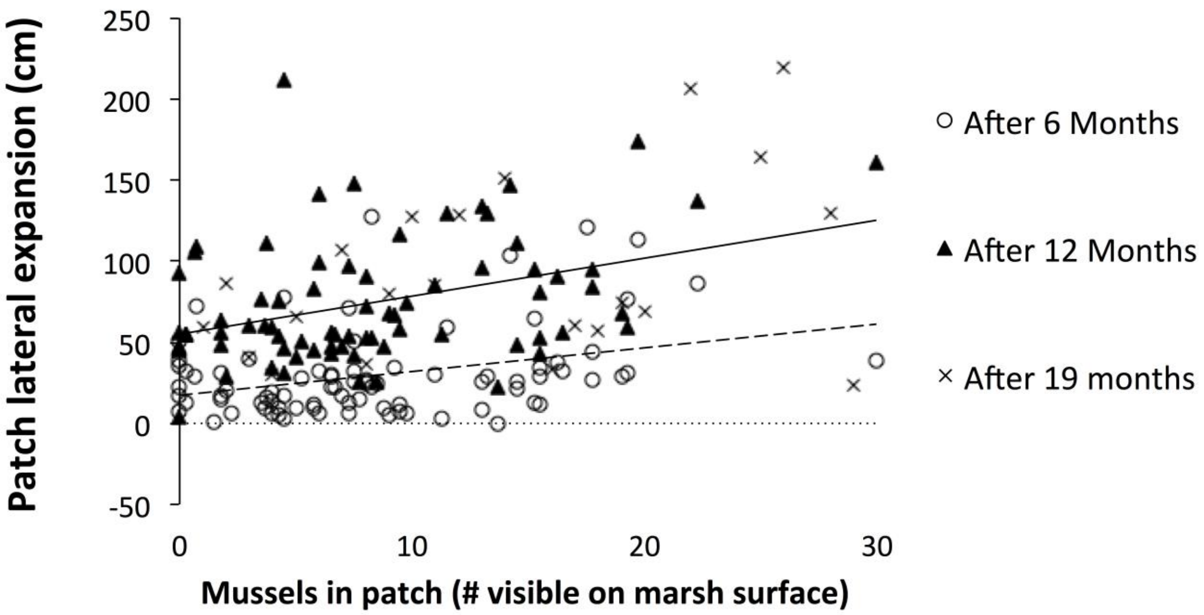

Supplementary Figure 4. Mussel density in natural patches increased the rate of cordgrass lateral expansion after 6 months (circles; linear regression, R^2= 0.14, P<0.0001) and 12 months (triangles; R^2= 0.12, P=0.001), but not after 19 months (crosses). Each point represents a unique cordgrass patch at each date.

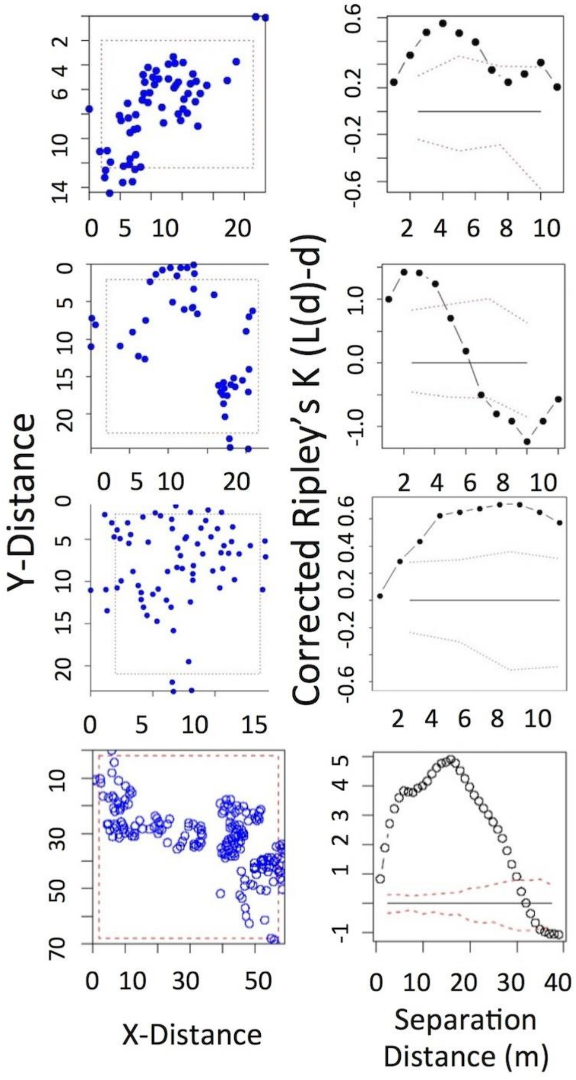

Supplementary Figure 5. Remnant patches vary in their spatial distribution within Sapelo Island die-off areas. Visual diagram of GPS-derived locations of remnant patches within four die-off areas (on left) and the corresponding Corrected Ripley's K values (on right) to describe the spatial distribution of those patches. Ripley's K values significantly greater than 0 (i.e. above the red 95% confidence interval lines) indicate remnant patches are clustered, while values significantly less than 0 indicate patches are over-dispersed at a given spatial scale.

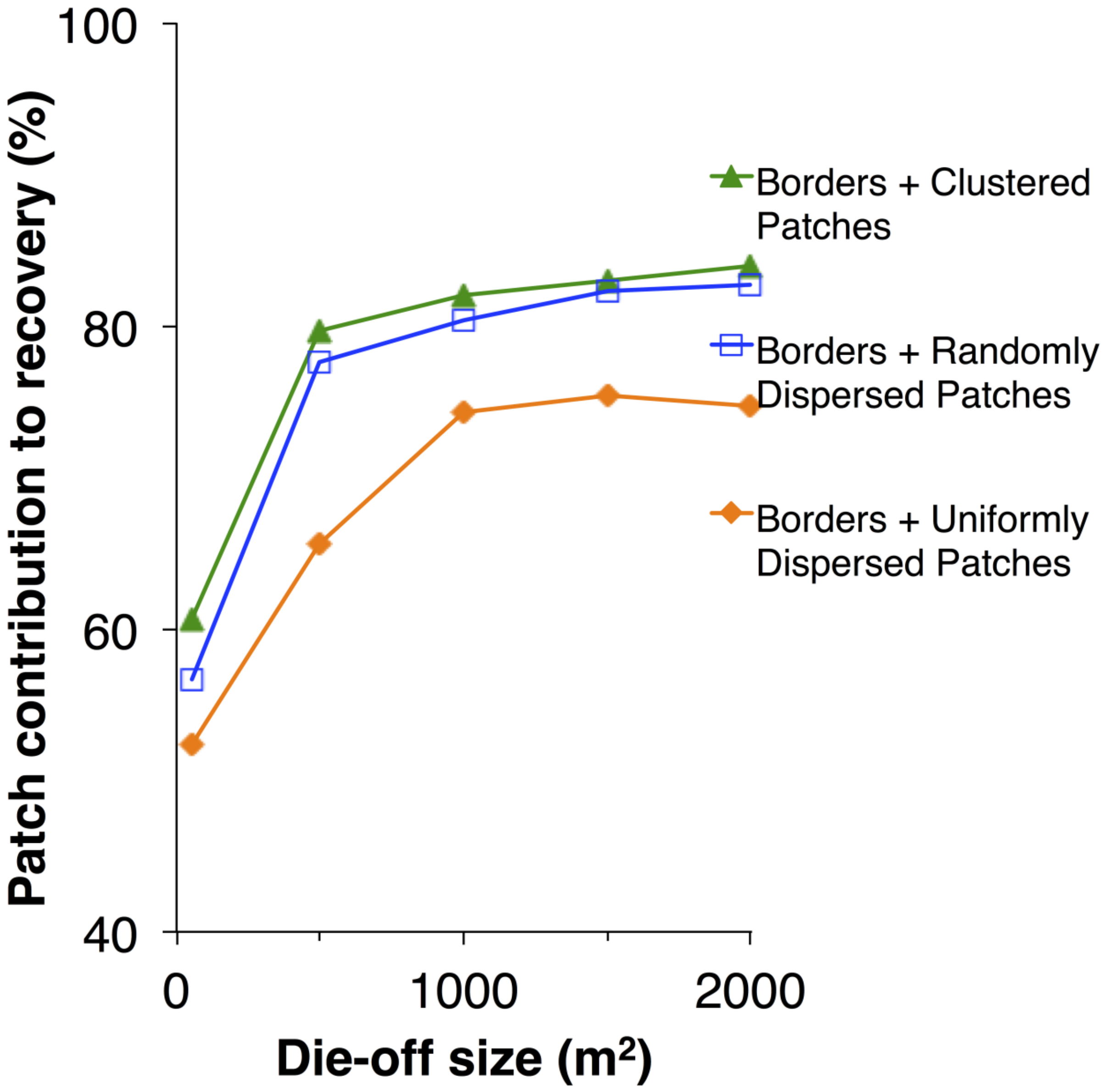

Supplementary Figure 6. Proportional contribution of patches to marsh recovery as a function of die-off size and patch dispersion. Data are shown as the mean of 3 simulation runs for each die-off size and patch dispersion combination. Results from Border Only and Border + Non-Dispersed Patch simulations are not shown as patches do not contribute to recovery in either of these scenarios.

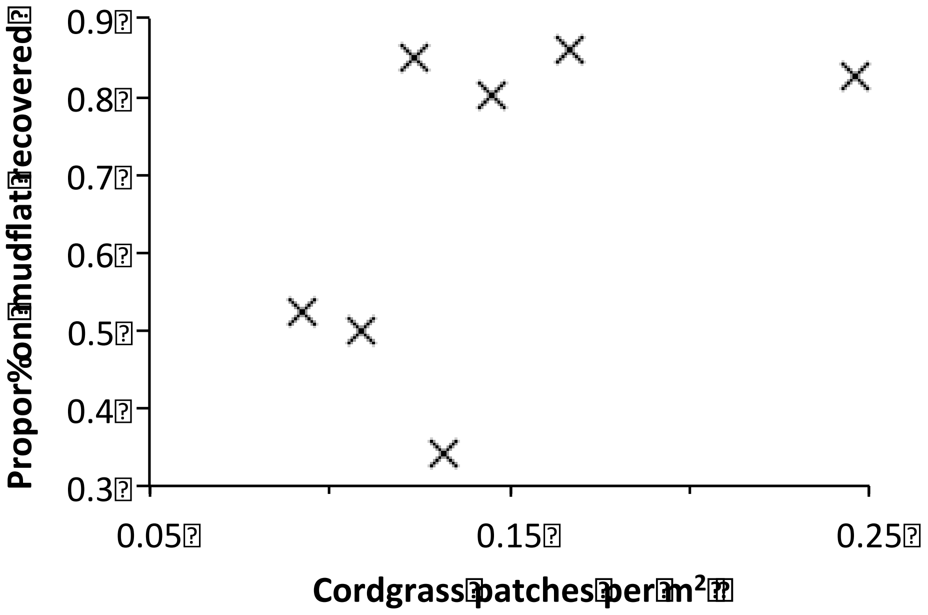

Supplementary Figure 7. Proportional change in drought-induced die-off mudflat area from June 2012 to July 2015 with increasing density of surviving cordgrass patches (linear regression: R^2=0.29, P=0.2). Data are from seven Sapelo Island die-off mudflats. Please note that die-offs with more than 0.15 patches per m^2 are nearly fully recovered after only 3 years.

Supplementary Table 1. Patterns in cordgrass die-off and survival along the southeastern US coast at the conclusion of a severe drought. Summary of cordgrass die-off spatial extent and the distribution of remnant cordgrass patches surviving in die-off areas associated with mussels in Georgia and South Carolina salt marshes. The mean and range of measurements are shown for all mudflats observed within each marsh sites.

Site (Latitude, Long)	# Die-offs	Die-off area m^2	% Die-off vegetated	Aspect ratio of die-off	Total remnant patch area, m^2	% Patches with mussels
Measurement displayed	*Total*	*Sum (range)*	*Mean (range)*	*Mean (range)*	*Mean (range)*	*Mean (range)*
Charleston, SC 32°46'46.14"N, 79°57'54.07"W	5	1688 (30-759)	2.0 (0.0 - 3.5)	0.67 (0.55-0.83)	0.016 (0 – 0.053)	50 (17- 83)
Folly Beach, SC 32°46'8.30"N, 79°58'18.94"W	7	6763 (100-2880)	1.8 (0.0 - 6.9)	0.55 (0.36-1)	0.014 (0 - 0.029)	80 (23- 100)
West Ashley, SC 32°46'25.36"N, 80° 0'4.24"W	3	7646 (625-1351)	3.1 (1.0 - 7.6)	0.70 (0.31-1)	0.022 (0.014- 0.031)	91 (76- 100)
Seabrook Isl., SC 32°34'57.07"N, 80°10'29.65"W	6	6070 (120-3750)	0.5 (0 .0 - 1.3)	0.33 (0.17-0.69)	0.018 (0- 0.015)	63.8 (75- 100)
Port Royal, SC 32°23'18.16"N 80°46'5.93"W	8	11259 (21-7350)	1.2 (0.0 - 0.7)	0.48 (0.16- 1)	0.014 (0- 0.056)	87.5 (75-100)

Fort Pulaski, GA 32° 1'25.50"N, 80°55'25.35"W	11	2563 (180- 435)	4.4 (4.2 - 4.9)	0.52 (0.17-1)	0.034 (0- 0.052)	53.8 (0- 100)
Sapelo Isl., GA, Oakdale marsh 31°24'26.11"N, 81°17'24.97"W	6	1759 (44- 823)	6.6 (1.4-10.4)	0.50 (0.21-0.80)	0.097 (0.023- 0.17)	64.8 (9- 100)
Sapelo Isl., GA Lighthouse marsh 31°23'29.54"N, 81°16'32.82"W	3	2438 (151- 1990)	7.7 (7.1 – 17.1)	0.51 (0.24- 0.91)	0.161 (0.09- 0.25)	86.2 (75- 100)
Jekyll Isl., GA 31° 5'27.68"N, 81°29'20.49"	4	11808 (90- 5808)	1.2 (1.1 - 1.6)	0.36 (0.19- 0.77)	0.046 (0.036- 0.054)	67.3 (29-98)

Supplementary Table 2. Effects of elevation above mean sea level, number of mussels within a patch, and average snail density on remnant *Spartina* patch areal expansion (change in cm^2) 6, 12 and 19 months after the conclusion of a severe drought. F-values and p-values are derived from linear mixed effects models run using the lme4 package in R. Marsh platform site was treated as a random factor in these analyses. Patches that had merged with other cordgrass patches or cordgrass bordering die-off areas were not monitored after 12 and 19 months, resulting in fewer patches being included in these analyses.

	After 6 months (79 patches monitored)		After 12 months (71 patches monitored)		After 19 months (64 patches monitored)	
Predictor variable	F-value	P-value	F-value	P-value	F-value	P-value
Elevation	17.8	**0.0001**	13.7	**0.0005**	6.2	**0.02**
# Mussels assoc. with patch	20.2	**<0.0001**	17.7	**0.0001**	0.8	0.37
Snails per $0.25m^2$	6.8	**0.01**	1.3	0.25	1.2	0.28
Elevation* Mussels	0.2	0.66	3.5	0.07	4.0	**0.05**
Elevation*Snails	2.7	0.10	0.3	0.60	0.2	0.68
Mussels*Snails	8.3	**0.005**	2.9	0.09	0.3	0.56
Elevation*Mussels*Snails	0.1	0.73	<0.1	0.99	1.4	0.24

Supplementary Table 3. Characteristics of Sapelo Island, GA die-off areas that were monitored in this study. Data collected from these areas were used to parameterize the cellular automaton model.

Site and ID#	Die-off area (m^2)	Die-off border perimeter (m)	# Cordgrass patches not assoc. with mussels	# Cordgrass patches assoc. with mussels	Initial patch cover, %
Oakdale 2	22.9	27.7	0	1	4.6
Oakdale 1	37.2	25.2	1	5	2.6
Oakdale 3	59.3	32.6	4	7	6.92
Lighthouse 3	134.8	50.3	0	22	10.0
Lighthouse 1	311.4	80.3	18	55	3.7
Oakdale 5	372.7	103.0	43	4	10.6
Oakdale 4	380.2	95.2	31	30	16.3
Oakdale 6	534.4	143.4	4	21	7.3
Lighthouse 2	1929.0	387.6	31	153	15.4
